# CD44 Binding to Hyaluronic Acid Is Redox Regulated by a Labile Disulfide Bond in the Hyaluronic Acid Binding Site

**DOI:** 10.1371/journal.pone.0138137

**Published:** 2015-09-17

**Authors:** Helena Kellett-Clarke, Monika Stegmann, A. Neil Barclay, Clive Metcalfe

**Affiliations:** The Sir William Dunn School of Pathology, University of Oxford, Oxford, United Kingdom; Institute for Virus Research, Laboratory of Infection and Prevention, JAPAN

## Abstract

CD44 is the primary leukocyte cell surface receptor for hyaluronic acid (HA), a component of the extracellular matrix. Enzymatic post translational cleavage of labile disulfide bonds is a mechanism by which proteins are structurally regulated by imparting an allosteric change and altering activity. We have identified one such disulfide bond in CD44 formed by Cys77 and Cys97 that stabilises the HA binding groove. This bond is labile on the surface of leukocytes treated with chemical and enzymatic reducing agents. Analysis of CD44 crystal structures reveal the disulfide bond to be solvent accessible and in the–LH hook configuration characteristic of labile disulfide bonds. Kinetic trapping and binding experiments on CD44-Fc chimeric proteins show the bond is preferentially reduced over the other disulfide bonds in CD44 and reduction inhibits the CD44-HA interaction. Furthermore cells transfected with CD44 no longer adhere to HA coated surfaces after pre-treatment with reducing agents. The implications of CD44 redox regulation are discussed in the context of immune function, disease and therapeutic strategies.

## Introduction

CD44 is an abundant, highly glycosylated transmembrane protein present on many leukocytes. The primary function of CD44 is to bind hyaluronic acid (HA) and mediate interactions between leukocytes and the extracellular matrix. One characteristic of CD44 is that it exists in many isoforms due to alternative splicing and different glycoforms and these control the HA binding function of CD44 [1]. CD44 is linked to many diseases, for example rheumatoid arthritis [2], cancer metastasis [3] and systemic lupus erythematosus [4]. Targeting CD44 with therapeutics has shown potential in rheumatoid arthritis [5,6] and various methods of modulating the CD44-HA interaction have been assessed in cancer therapy [7,8]. Regulation of CD44 activity is clearly an important and complex system with huge therapeutic potential. CD44 was revealed in recent screens for membrane proteins containing labile disulfide bonds [9] suggesting a role for the extracellular redox environment in regulation of CD44 activity.

Disulfide bonds formed between cysteine residues play an important role in the stabilisation and organisation of proteins but it is now apparent that a subset of these bonds are labile and can function as redox switches [10]. Changes in the redox microenvironment through the secretion of thiol reductase and protein disulfide isomerase (PDI) enzymes can impart post translational allosteric structural changes in proteins [11] resulting in modulation of protein and cellular function. Post translation control of disulfide bonds is essential for maintaining haemostasis with many blood proteins under redox control [12]. For example, in the early stages of thrombus formation activated platelets secrete PDI which reduces two disulfide bonds in the integrin αIIbβ3 on the platelet surface. This results in allosteric switching of fibrinogen cross linking and inhibition of PDI inhibits thrombus formation (redox control of platelet function is reviewed in [13]).

There is evidence that post translational control of disulfide bond topology also plays a role in regulation of the immune system. Activation of T cells results in up regulation of thioredoxin-1 (Trx1), a ubiquitous thiol reductase enzyme [14], and results in increased thiols at the T cell surface [15,16], which can be inhibited with thiol reductase inhibitors [15]. Activated dendritic cells also secrete Trx1 [17] which can reduce disulfide bonds at the surface of both the dendritic cells and co cultured T cells [18]. This surface reduction is inhibited by introducing regulatory T cells [18]. Immunisation of mice leads to an increase in free thiols at the surface of the leukocytes [14] as does LPS induced endotoxemia [9]. A key question towards understanding the role of redox reduction of surface proteins in immune responses is which proteins contain the reduced disulfide bonds?

This was addressed for the first time when we recently developed a mass spectrometry based screen to identify proteins on the cell surface of leukocytes that contain a labile disulfide bond [9]. More than 50 different proteins were identified on the cell surface of a mouse 2B4 T-cell hybridoma that potentially contain such bonds. To demonstrate the efficacy of the screen we followed up CD132, the common γ signalling chain of the interleukin-2 (IL-2) family of cytokine receptors with functional assays and showed that function of the IL-2 receptor could be controlled through reduction of a labile disulfide bond at the CD132/IL-2 binding interface [19].

CD44 was consistently found in these screens for membrane proteins with labile disulfide bonds and herein we explore the HA binding function of CD44 in response to protein redox state. We show a novel mechanism by which CD44 can be regulated by enzymes known to be secreted into the extracellular environment during inflammation. The implications for the function of CD44 and other HA binding proteins of the link module family are discussed together with possibilities for new avenues of manipulating these interactions for therapeutic purposes.

## Materials and Methods

Human and mouse CD44 Fc proteins were purchased from R&D systems. Biotinylated rat anti-mouse/human CD44, (clone IM-7) and biotinylated goat anti rat IgG were purchased from Abcam. HRP-conjugated anti human and mouse Fc and FITC and DyLight 800 conjugated avidin were purchased from AbD Serotec. Peptide-N-Glycosidase F (PNGase F) was purchased from New England Biolabs. Deuterated N-ethylmaleimide (ethyl-D5) was from Cambridge Isotopes. Everything else was purchased from Sigma-Aldrich.

### TCEP-HCl reduction and differential thiol labelling of the GFETCR peptide of CD44 on the surface of 2B4 cells

Murine T cell hybridoma cells, 2B4 Saito [20] (~1 x 10^8^ cells) were reduced with TCEP-HCl (2.5 mM in PBS containing 1% BSA), reacted with maleimide-PEO2-Biotin (MPB), the biotinylated cell surface glycoproteins purified, digested with PNGase F and trypsin and analysed by MS [9]. Non reduced MPB labelled cells were used as a control to determine background levels of free cysteines on the cell.

### Kinetic trapping of disulfide bond reduction in hCD44-Fc chimeras

hCD44 Fc-chimera (5 μg in PBS) was added to 10kDa 500 μl centrifugal concentrators (Vivacon 500, Sartorious). Two samples were reduced with TCEP-HCl (100 μl of 2.5 mM in PBS for 15 minutes at 4°C) and Trx1 (100 μl of 1 μM Trx1 supplemented with 100 nM TR1 and 200 μM NADPH for 90 minutes at 37°C). A non-reduced control sample was treated with 100 μl of PBS at 37°C for 90 minutes. After washing, reduced disulfides were kinetically trapped by alkylating with NEM (100 μl 1 mM in PBS for 30 minutes at 4°C). After washing the chimeras were denatured (10 mM TCEP-HCl in 8M urea for 1 hour at room temperature), washed and any remaining cysteines alkylated with D5-NEM (100 μl of 1 mM in PBS for 30 minutes at 4°C). The samples were deglycosylated and trypsin digested [9].

### Mass spectrometry

After desalting on a C18 micro column, the samples were resuspended in 0.1% formic acid containing 2% acetonitrile and analysed on a Ultimate 3000 UHPLC (Dionex) coupled to a QExactive mass spectrometer (Thermo Fisher Scientific). Samples were injected directly on an in-house packed 25 cm C18 (Bishoff 3 micron bead diameter) column. Separation of peptides was achieved with the following gradient 5–30% buffer B over 90 min, 30–55% buffer B over 20 minutes and 98% buffer B for 5 minutes (buffer A: 0.1% formic acid, buffer B: 0.1% formic acid in acetonitrile) at a flow rate of 300nl/minute. Data were acquired in a data-dependent mode, automatically switching from MS to collision induced dissociation MS/MS on the top 20 most abundant ions with a precursor ion scan range of 350–1650 m/z. Full scan MS spectra were acquired at a resolution of 70,000 and MS/MS scans at 17,500 at a target value 3 x 10^6^ and 1 x 10^5^ ions respectively. Dynamic exclusion was enabled with exclusion duration of 40 seconds.

### Semi-quantitative label free analysis of GFETCR from 2B4 cells

Label free analysis was performed (Progenesis QI for Proteomics software, Nonlinear Dynamics) and aligned precursor features were searched with Mascot. MS/MS spectra were searched against the UniProt mouse database. Precursor mass tolerance was 10 ppm and a fragment ion tolerance was 0.02 Da with a minimum ion score of 20. The precursor ion charge state was 2+, 3+ and 4+. Variable modifications were defined as deamidation on asparagine and glutamine, oxidation on methionine and alkylation with MPB or IAA on cysteine. The enzyme specificity was set to trypsin with a maximum of two missed cleavages. All searches were performed against a concatenated target/decoy database, providing an empirical false discovery rate (FDR) and results are reported at a 1% target/decoy FDR for both peptides and proteins. Aligned chromatography features identified as MPB and MPB+H_2_O alkylated GFETCR in both control and TCEP-HCl reduced samples were visually compared.

### Quantitative data analysis of disulfide bond reduction of kinetically trapped hCD44-Fc

The data files from all of the mass spectrometry runs were combined and searched against the human Swiss-Prot database using Peaks 7 proteomics studio (Bioinformatics Solutions Inc. On, Canada). Precursor mass tolerance was 10 ppm and a fragment ion tolerance was 0.01m/z with up to three missed trypsin cleavage sites per peptide allowed. Variable modifications were defined as deamidation on asparagine and glutamine, oxidation on methionine and alkylation with NEM or D5-NEM (and hydrolysed variants) on cysteines. *de-novo*, peaks-db, SPIDER and peaks PTM algorithms were sequentially used to search against a concatenated target/decoy database, providing an empirical FDR and results are reported at a 1%t target/decoy FDR for both peptides and proteins. 2+ ions and retention time windows were extracted for peptides containing cysteine residues from the hCD44 hyaluronic binding domain (HABD) and the Fc region of the chimera. Precursor ion areas for NEM and D5-NEM alkylated (and hydrolysed variants) peptides were extracted using MS1 filtering in Skyline. The reduction of each cysteine is calculated from the following ratio (total NEM area/total D5-NEM area) where total NEM area and total D5-NEM area are the sum of normal and hydrolysed forms of NEM and D5-NEM (plus any other variants such as oxidised methionine and deamidation) and normalised against the control ratio for each peptide.

### Generation of a CD44 transfected CHO cell line

The coding sequence was amplified from a full length cDNA using the high fidelity polymerase pfu Ultra AD (Agilent) with the following primers:

hCD44–14 MluI F 5’ GCGTAACGCGTCCCGGACACCATGGACAAG

hCD44 1146* NotI R 5’ CGGCGGCCGCTTA CACCCCAATCTTCATGTCCAC

Segments of the primers that generate restriction sites are underlined and the stop codon is denoted by italics in the reverse primer.

The amplified product was cloned into the vector pHR Sin, based on the HIV retrovirus. 293T cells were transiently transfected with pHR Sin plasmids together with pMD.G and p8.91 [21] in 6-well plates using Genejuice (Merck, Darmstadt, Germany) according to the manufacturer’s instructions. Growth media had been changed immediately prior to transfection with DMEM supplemented with 10% FCS. Supernatant was harvested at 48–72 hours post-transfection passed through a 0.45 micron filter to remove cell debris. Transduction was achieved by adding virus-like particles in 2 ml of supernatant to 2x10^5^ CHO K1. Cells were incubated overnight before the supernatants were replaced with fresh growth medium. After 3 days cells bearing an enhanced level of cell surface CD44 with concomitant elevated levels of HA binding (as determined by staining with fluorescein labelled HA) were isolated by FACS and maintained as a line.

### CD44-Fc HA plate binding assays

ELISA plate wells were coated with 5mg/ml HA in carbonate-bicarbonate coating buffer, pH 9.6 for 18 hours at 4°C, washed with PBS containing 0.05% tween-20 and blocked with 1% BSA for 3 hours at room temperature. Human (h) and mouse (m) CD44-Fc protein were diluted to 5mg/ml in PBS and reduced with either TCEP-HCl (2.5 mM for 20 minutes at 4°C) or Trx1 (100 μl of 1 μM Trx1 supplemented with 100 nM TR1 and 200 μM NADPH for 90 minutes at 37°C) after which reduced chimeras were alkylated by 5 mM NEM for 30 minutes at 4°C. A non reduced control samples were treated with 5 mM NEM only. The stock solutions were serially diluted to concentrations of 5000, 2500, 1250, 625, 313, 156, 78, 39, 20, 10, 5 and 2.5ng/ml (plus 5mg/ml to determine maximal binding), added to HA coated ELISA plates in triplicate and left to equilibrate at room temperature for 1 hour. Bound CD44-Fc was probed with HRP-conjugated anti-human Fc or HRP conjugated anti-mouse Fc with colorimetric quantitation using 3,3',5,5'–tetramethylbenzidine substrate, quenched with 5% H_2_SO_4_ and optical densities recorded at 450 nm. Binding isotherms were constructed and normalised by plotting mean absorbances for each CD44-Fc concentration as a fraction of the maximal binding and equilibrium dissociation constants (*K*_*D*_) determined by fitting a one site binding model to the data in Graphpad Prism 6.0 software. Quoted *K*_*D*_s and the final figures are the average of three independent experiments.

### Staining of reduced cysteines on hCD44 transfected CHO cells and western blot for CD44

CHO hCD44+ cells (~1x10^7^ each sample) were washed twice with ice cold PBS and reduced with either TCEP-HCl (1ml of 2.5 mM in PBS/1%BSA for 15 minutes at 4°C) or Trx1 (1ml of 100 μl of 1 μM Trx1 supplemented with 100 nM TR1 and 200 μM NADPH for 90 minutes at 37°) after which they were washed x2 with ice cold PBS and resuspended in 100uM Dylight-800-maleimide in PBS for 30 mins at 4°C to label reduced cell surface disulfide bonds. Non reduced cells were labelled as a control. After washing x5 with ice cold PBS the cells were lysed in 1ml of 1% digitonin for 30 minutes on ice. The lysates were cleared of cell debris by centrifugation at 11,000g for 20 minutes, and a sample mixed 1:1 with non-reducing SDS-PAGE sample loading buffer, heated to 95°C for 5 minutes and loaded onto Novex 4–12% bis-tris SDS PAGE gels, resolved in SDS-MES buffer at 180V and visualised on a LICOR™ Odyssey imaging system at 700 nm for the protein molecular weight markers and 800 nm for the labelled samples. The gel was transferred on nitrocellulose membrane and probed with biotinylated IM-7 CD44 specific monoclonal antibody that was detected with streptavidin-Dylight-680 and visualised on a Licor Odyssey imaging system. Scans of the gel and the blot were aligned via the protein molecular weight standards using Licor image studio software.

### Immunoprecipitation of reduced CD44 from hCD44 transfected CHO cells

Dynabead-M280 streptavidin coated magnetic beads (1 μg per immunoprecipitation) saturated with biotinylated IM7 CD44 antibody (10 μg per immunoprecipitation) were added to lysates and rotated at 4°C for 4 hours. The beads were isolated, washed x4 with ice cold 0.1% digitonin in PBS and heated at 95°C for 10 minutes in 50 μl of 100 mM glycine-HCl, pH 2.8. 10 μl of the eluent was mixed 1:1 with non-reducing SDS-Page sample loading buffer, centrifuged at 11,000g for 10 minutes, loaded onto Novex 4–12% bis-tris SDS PAGE gels and resolved in SDS-MES buffer at 180V and visualised on a LICOR™ Odyssey imaging system at 700 nm for the protein molecular weight markers and 800 nm for the labelled samples.

### hCD44 transfected CHO cell adhesion assay

ELISA plate wells were coated with 5mg/ml HA in carbonate-bicarbonate coating buffer, pH 9.6 for 18 hours at 4°C, control HA free wells were treated with coating buffer alone. Plates were washed with PBS containing 0.05% Tween 20 and blocked with 1% BSA for 3 hours at room temperature. hCD44 transfected CHO cells were fluorescently labelled using a Vybrant® Cell Adhesion Assay Kit (Life technologies). Cells (5x10^7^) were reduced with TCEP-HCl (1ml of 2.5 mM in PBS/1%BSA for 15 minutes at 4°C) or Trx1 (1ml of 100 μl of 1 μM Trx1 supplemented with 100 nM TR1 and 200 μM NADPH for 90 minutes at 37°) after which they were washed twice in ice cold PBS and alkylated with 5 mM NEM in PBS for 30 mins at 4°C. Non reduced cells were also alkylated as a control. After resuspension in DMEM supplemented with 5% FCS, 1x10^6^ cells were added to HA coated and control wells respectively, each condition in quintuplicate. Cells were left to adhere for 45 minutes at 37°C after which wells were carefully washed with PBS to remove non adherent cells. Total fluorescence was recorded at 517 nm and data from each individual well was normalised to the mean total fluorescence of the five non reduced hCD44+CHO cells binding HA coated wells, the mean of which was defined as 100% binding. In order to represent the variability of the binding assay the results are graphed as composites of 15 wells per experimental condition (five wells per condition form three independent experiments) and significance of reduction was determined by applying non paired T tests to each condition relative to the control.

## Results

### CD44 contains disulfide bonds that are potentially labile

**Fig 1:**
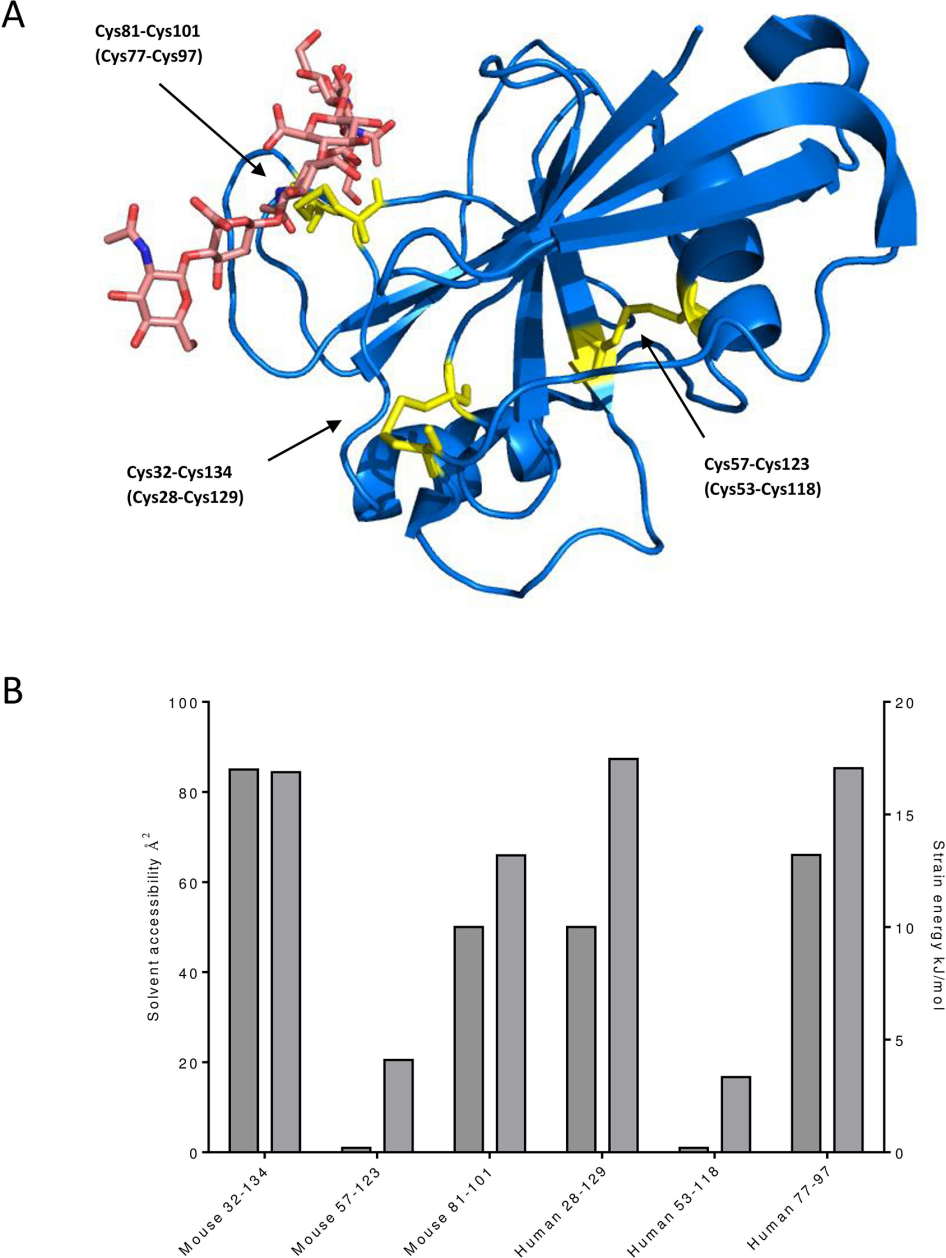
(A) Crystal structure of the HABD of murine CD44 in complex with an HA 8-mer (adapted from PDB ID:2JCQ). The protein backbone is represented as a blue cartoon. The HA 8-mer is shown as pink sticks. Disulfide bonds are shown as yellow sticks and the cysteine residues from which they are formed are numbered according to their position in the mouse CD44 sequence (human numbering is shown in parenthesis). (B) For each disulfide bond dark grey bars show solvent accessibility and light grey bars show bond strain energy calculated from the PDB coordinates (PDB ID:2JCQ for mouse and 1UUH for human, the coordinates for HA were removed from 2JCQ prior to analysis) **using** (http://powcs.med.unsw.edu.au/research/adult-cancer-program/services-resources/disulfide-bond-analysis-tool/disulfide-bond).

Potentially labile disulfide bonds can be characterised and identified by their configuration (labile bonds are normally–RH Staple,–LH Hook or–/+RH Hook), the strain energy of the covalent bond between the sulphur atoms and accessibility to reductases (defined as the solvent accessibility of the cysteines making up the bond) [11]. The crystal structure of the N-terminal globular HABD of murine CD44 in complex with a HA octosaccharide [22] is shown in Fig 1A. The HABD consists of the link module, typical of many hyaladherins which contains a shallow HA binding groove. The link module contains two conserved disulfide bonds and Cys57-Cys123 (Cys53-Cys118 in human) which stabilises the core and (Cys81-Cys101 (Cys77-Cys97 in human)) which is located at the bottom of the HA binding groove and stabilises it and is in contact with bound HA. There is a third disulfide bond Cys32-Cys134 (Cys28-Cys129 in human) which stabilises an area of the HABD formed by C and N terminal extensions to the link module.

Structural analysis of the disulfide bonds using the bioinformatics methods of [23] in the HABD of CD44 (Fig 1B) reveals two potentially labile disulfide bonds. Both the HA binding groove disulfide and (Cys32-Cys134 (Cys28-Cys129 in human) in the link module extension have solvent accessibility in excess of 50 Å^2^. Furthermore they have high bond strain energy and potentially labile configurations of–LH Hook and–RH Staple respectively. The third disulfide bond, Cys57-Cys123 (Cys53-Cys118 in human)), is buried in the globular core of the HABD and has low strain energy and a non-labile spiral configuration.

### A disulfide bond in CD44 is labile on the human and mouse leukocyte surface

**Fig 2:**
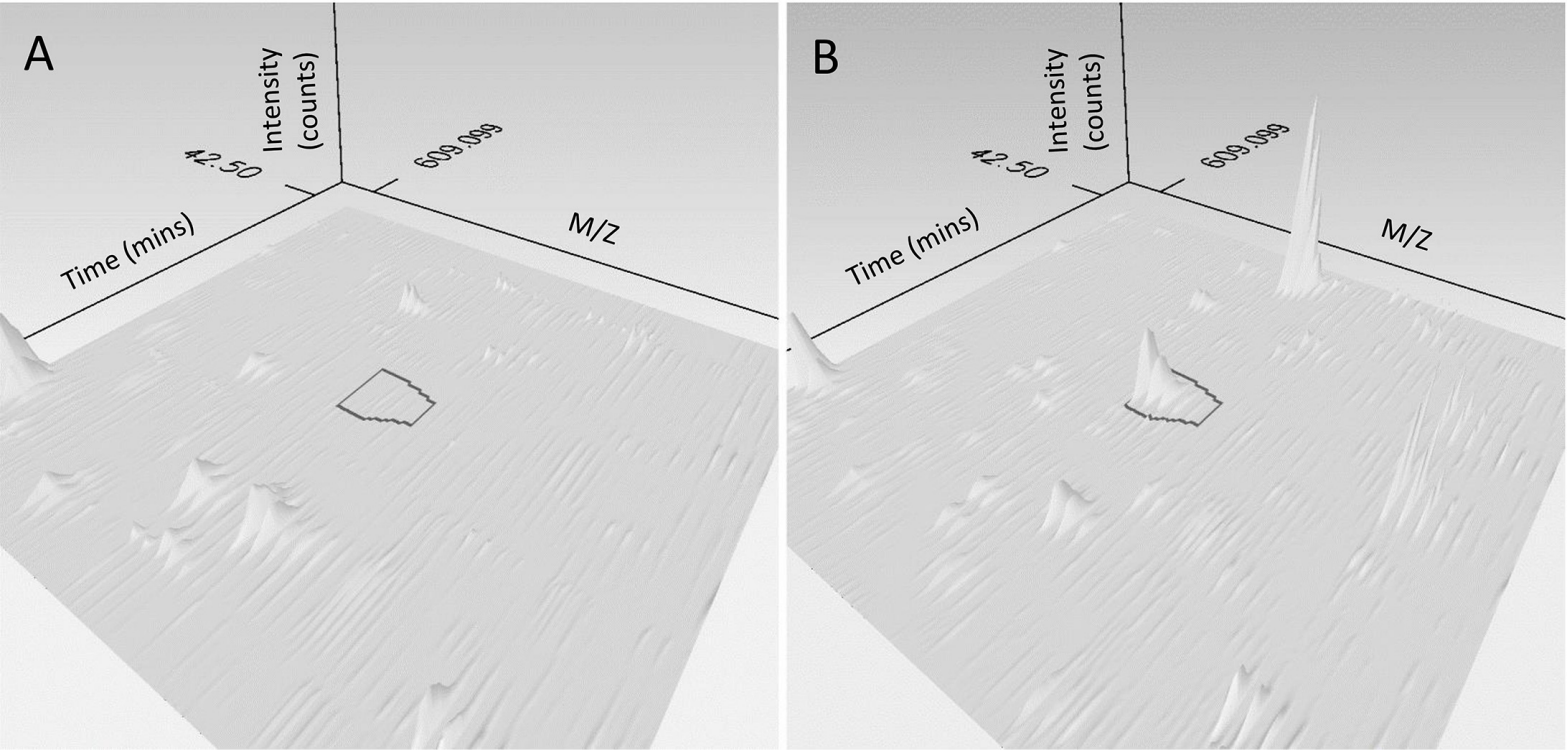
Aligned 3D total ion current chromatograms using Progenesis QI in the area of chromatographic space where MPB labelled GFETCR elutes for (A) control 2B4 cells and (B) TCEP-HCl reduced 2B4 cells. The peak corresponding to MPB labelled GFETCR is outlined in black and is visibly larger in the TCEP-HCl reduced cells. Other peptides are seen in both the control and TCEP-HCl reduced samples, ones with increased area after TCEP-HCl treatment suggests they are from proteins containing labile disulfide bonds.

Previously as part of a large scale mass spectrometry screen we identified CD44 on the surface of a mouse T cell hybridoma 2B4 to contain a reduced, and therefore potentially labile disulfide bond [9]. Further analysis of the data reveals the peptide GFETCR, containing Cys81 from the disulfide bond that stabilises the HA binding to be alkylated with MPB when CD44 is purified from TCEP-HCl or human thioredoxin-1 (Trx1) reduced cells. This indicates that this cysteine originated from a labile disulfide bond that was reduced when the cells were treated. On untreated control cells the Cys81 was not labelled with MPB therefore MPB alkylation and hence disulfide bond reduction appears to be dependent upon exogenous reducing agents. These screens are non-quantitative and provide no information on the extent of reduction of the labile bond at the cell surface therefore a more quantitative mass spectrometry workflow was designed. As the amount of cysteine biotinylation by MPB is proportional to the amount of reduction of the disulfide bond then if the disulfide bond is labile and therefore MPB labelled, then more protein from reduced cells will be captured on avidin beads than from control cells. Progenesis QI software suite was used to align the total ion current chromatograms (TICs) from control and TCEP-HCl reduced 2B4 hybridoma cells. Fig 2 shows the region of the TICs where MPB labelled GFETCR elutes in both the control (Fig 2A) and the TCEP-HCl reduced (Fig 2B) cells. The peak area of GFETCR is much greater in the TCEP-HCl reduced sample than in the control where the peak for GFETCR does not rise above background levels. Other local spectral features unrelated to CD44 are seen in both the control and TCEP-HCl reduced samples, and some of the intensities are greater in the TCEP-HCl reduced sample, suggesting the peptides are from other proteins enriched through MPB labelling of redox labile disulfide bonds. A second cysteine containing peptide TEAADLCQAFNSTLPTMDQMK which contains Cys57 from the disulfide bond buried deep in the link module is consistently identified in the mass spectrometry screens but is never labelled with MPB, suggesting that it forms a disulfide bond in CD44 that is stable towards reduction, as was predicted by the structural analysis. Combining these observations show that the disulfide bond in the HA binding groove in CD44 is labile on the surface of the 2B4 T cell hybridoma, being readily reduced by TCEP-HCl. Subsequently this disulfide was observed to be labile under many differing conditions in screens from several other mouse and human leukocytes as summarised in Table 1. All subsequent experiments were performed on human CD44 and therefore human numbering will be used from this point onwards.

**Table 1:** Cell types and treatments for which the allosteric disulfide has been observed.

Species	Cell Type	Treatment	HA peptide	Labile?	Globular peptide	Labile?^c^
Mouse	2B4	Control	GFETCR	No	TEAADLCQAFNSTLPTMDQMK	No
		TCEP-HCl		Yes		No
		Trx1		Yes		No
		GILT^a^		Yes		No
Human	Platelets	Control	ALSIGFETCR	No	TEAADLCK	-
		TCEP-HCl		Yes		-
		DTT		Yes		-
Human	MEG-01	Control	ALSIGFETCR	No	TEAADLCK	-
		TCEP-HCl		Yes		No
Human	Namalwa	Control	ALSIGFETCR	No	TEAADLCK	-
		TCEP-HCl		Yes		-
Human	PBMC	Control	ALSIGFETCR	Yes	TEAADLCK	No
		TCEP-HCl		Yes		No
		SEA+IL-2^b^		Yes		No

^a^ gamma-interferon-inducible lysosomal thiol reductase.

^b^ MLR supplemented with staphylococcal enterotoxin A super antigen.

^c^–is not detected in the mass spectrometry run.

### The disulfide bond in the HA binding groove of recombinant soluble CD44 is preferentially reduced

**Fig 3:**
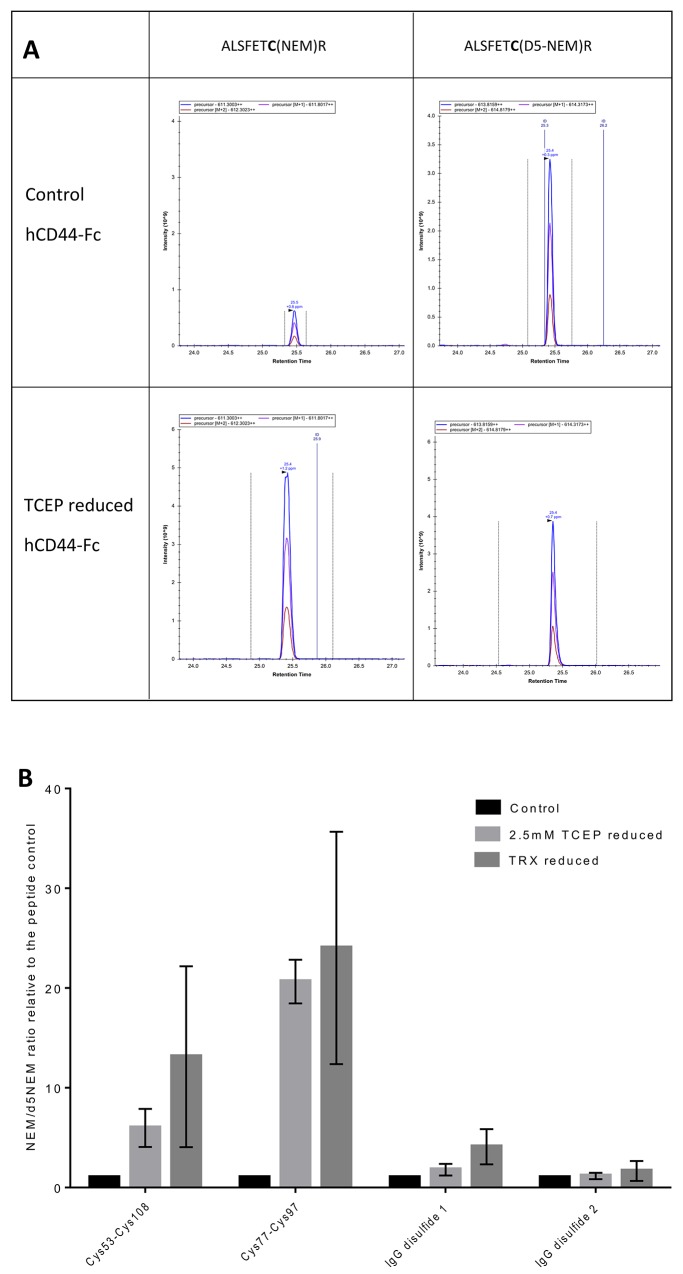
The Cys77-Cys97 disulfide bond is the most reactive in hCD44-Fc chimera after treatment with TCEP-HCl and Trx1 followed by kinetic trapping with NEM. (A) Extracted ion chromatograms for NEM and D5-NEM labelled ALSIGFETCR in non reduced control and TCEP reduced hCD44-Fc. In the control the NEM labelled peptide has a much smaller area than the D5-labelled peptide showing that the Cys77-Cys97 disulfide bond is predominantly oxidised. After reduction with TCEP the area of NEM labelled peptide is increased relative to D5-NEM labelled peptide indicating that the disulfide bond has been significantly reduced. Peak integration boundaries are shown as dotted lines and the times of any MS/MS switching events are indicated. (B) Summary of the ratio of the extracted ion chromatograms for NEM labelled to D5-NEM labelled peptides. Peptide ALSIGFETCR contains Cys77 is from the disulfide bond in the HA binding groove. TEAADLCK contains Cys53 and is from the disulfide buried in the core of the link domain. PEVTCVVVDVSHEDPEVK and NQVSLTCLVK are from the Fc region of the chimera. Columns represent the fold change of the NEM/D5-NEM ratio of each peptide for TCEP-HCl reduced chimera (medium grey), Trx1 reduced chimera (light grey) relative to non-reduced control chimera (dark grey). Mean and SEM of ratios from two reductions are shown.

A key factor in determining whether the reduction of the labile disulfide bond in CD44 has a functional effect is the extent to which it occurs. This was tested using samples of human Fc chimera fusion protein, hCD44-Fc which were reduced with either TCEP-HCl or Trx1 for 20 and 90 minutes respectively. The resultant redox state of hCD44-Fc was kinetically trapped by alkylating free cysteines liberated from disulfide bond reduction with NEM. A control sample of hCD44-Fc was alkylated with NEM without prior reduction to obtain background levels of free cysteines. The labelled fusion proteins were then denatured with urea and further reduced with TCEP-HCl and the remaining disulfide bonds in hCD44-Fc were alkylated with deuterium labelled NEM, D5-NEM. After deglycosylation with PNGase F, digestion with porcine trypsin, and LC-MS-MS analysis, Peaks-7 was used to search the data against the UniProt human protein database [24] and four high ranking cysteine containing peptides were chosen to quantify the reduction of the disulfide bonds. TEAADLCK (Cys53) and ALSIGFETCR (Cys77) from the hCD44 HABD plus PEVTCVVVDVSHEDPEVK (Cys144) and NQVSLTCLVK (Cys250) from each of the immunoglobulin domains of the Fc region of the fusion protein. Precursor ion areas were extracted for the 2+ ion of each modified peptide along with two topisomers, examples of extracted ion chromatograms for ALSIGFETCR before and after reduction with TCEP are shown in Fig 3A. The total area for each peptide for each condition was determined by adding the normal and hydrolysed forms of the NEM together. The amount of reduction is determined by ratio of NEM peptide areas to D5-NEM peptide areas and is summarised in S1 Table. For each of the two replicates the increase in the NEM/ D5-NEM precursor ion area ratios for a given peptide after reduction are normalised to the control (Table 2) and plotted in Fig 3B. After reaction with TCEP-HCl and Trx1 the NEM:D5-NEM ratio of ALSIGFETCR (Cys77-Cys97) increases >20 fold over the control whereas TEAADLCK (Cys53-Cys118) only increases to approximately 12 fold after Trx1 reduction and <10 fold after TCEP-HCl reduction. The Cys77-Cys97 disulfide bond from the HA binding groove is more reactive, thus more labile than the Cys53-Cys118 disulfide bond in the core of the link domain, corroborating the evidence seen in the cell surface screens. As expected, the peptides from buried disulfide bonds in the tightly folded immunoglobulin domains of the Fc region showed no significant reduction, and serve as an internal control. Both of the cysteines from the Cys28-Cys129 disulfide bonds do not reside in tryptic peptides that are compatible with mass spectrometry so their reduction state could not be determined.

**Table 2:** Summary of NEM/D5-NEM peptide ratios and fold changes relative to controls determined from the summed extracted ion chromatogram areas of the indicated peptides calculated in S1 Table.

	NEM/D5-NEM ratio						NEM/D5 NEM ratio change relative to control					
Peptide	Replicate 1			Replicate 2			Replicate 1			Replicate 2		
	Control	TCEP	Trx1	Control	TCEP	Trx1	Control	TCEP	Trx1	Control	TCEP	Trx1
ALSIGFETCR	0.05	1.21	1.89	0.15	2.81	1.88	1.00	22.83	35.66	1.00	18.45	12.37
TEAADLCK	0.06	0.47	1.31	0.24	0.99	0.98	1.00	7.88	22.19	1.00	4.07	4.05
NQVSLTCLVK	0.08	0.19	0.48	0.44	0.53	1.02	1.00	2.37	5.85	1.00	1.21	2.32
TPEVTCVVVDVSHEDPEVK	0.16	0.13	0.41	0.19	0.28	0.12	1.00	0.85	2.66	1.00	1.48	0.66

### Disulfide bond reduction of recombinant soluble CD44 inhibits HA binding

**Fig 4:**
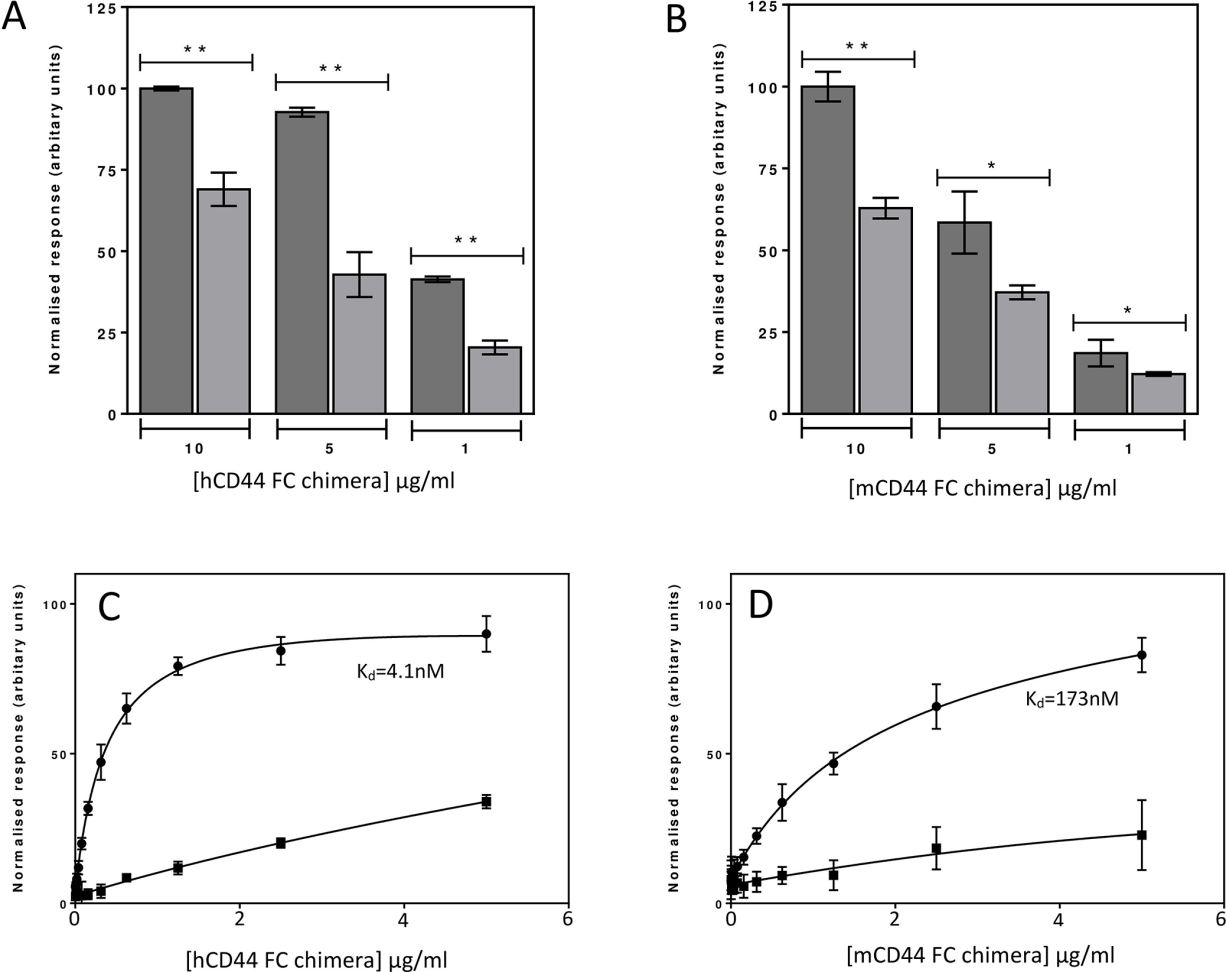
TCEP-HCl reduction of CD44 inhibits binding to HA. (A) hCD44-Fc and (B) mCD44-Fc binding to plates coated with 50 μg/ml HA. Columns represent non reduced control CD44-Fc (dark grey) and TCEP-HCl reduced CD44-Fc (light grey). Mean and SEM of peptide chromatographic areas from three experiments are shown, the differences between non reduced and TCEP-HCl reduced chimera: *, P < 0.05; **, P < 0.01. Titrations of Trx1 reduced (C) human CD44-Fc and (D) mouse CD44-Fc binding to plates coated with 50 μg/ml HA. For both mouse and human CD44-Fc titrations the binding curves represent non reduced control CD44-Fc (black circles) and Trx1 reduced CD44-Fc (black squares). Binding curves are constructed from data of three independent experiments and equilibrium dissociation constants were determined by fitting to a single binding site model.

To test the effect that reduction of the disulfide bond in the CD44 HA binding groove has on HA binding function we conducted binding experiments with plate bound HA. Both hCD44-Fc and mCD44-Fc were reduced as above with either TCEP-HCl or Trx1 and alkylated with NEM. Non reduced controls were alkylated without prior reduction. The binding of both hCD44-Fc (Fig 4A) and mCD44-Fc (Fig 4B) to plate bound HA was significantly inhibited by treatment with TCEP-HCl. For Trx1 reduction, which is more biologically relevant, a full titration was performed for hCD44-Fc (Fig 4C) and mCD44-Fc (Fig 4D). Combined data from three experiments gave equilibrium dissociation constants (K_D_) of 4.5 ± 0.41 nM for hCD44-Fc and 180 ± 2.7 nM for mCD44-Fc respectively. After reduction, binding to HA was almost completely inhibited. The HA binding groove disulfide bond is therefore not only labile but is also functional, acting as a redox sensitive switch controlling HA binding.

### CD44 can be reduced on the surface of transfected CHO cells

**Fig 5:**
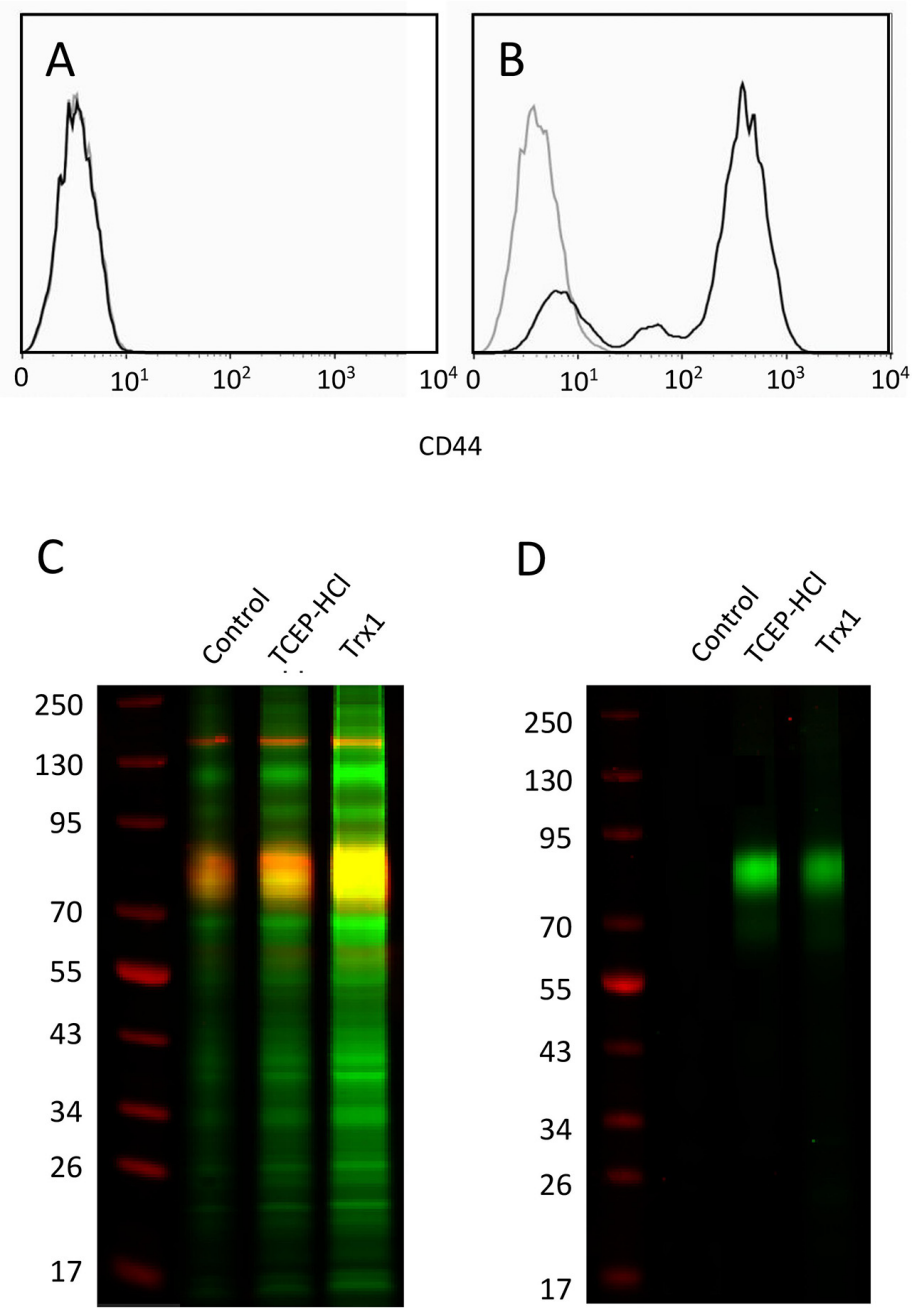
Levels of CD44 determined by flow cytometry analysis of (A) untransfected CHO K1 cells and (B) hCD44 transfected CHO cells. Light grey trace is isotype control, black trace is anti-CD44. (C) Non reduced SDS-PAGE gel showing reduced proteins on the surface of hCD44 transfected CHO cells after reduction (green bands) overlaid with a western blot of CD44 (red). (D) non reduced SDS-PAGE gel of eluted proteins which contain reduced disulfide bonds (green bands) from a CD44 immunoprecipitation of hCD44 transfected CHO cells that had been reduced with either TCEP-HCl or Trx1.

Mass spectrometry is an excellent tool to screen the cell surface proteome as a whole to determine which proteins contain labile disulfide bonds. However it is expensive and requires complex and lengthy sample preparation so is not practical to routinely check for reduction of a specific cell surface protein prior to cellular assays. To address this we developed a gel based assay to quantify CD44 reduction on the surface of CHO cells transfected with a full length hCD44 construct, which express high levels of CD44 on their surface (Fig 5A and 5B). CHO-hCD44 cells were reduced with TCEP-HCl and Trx1 under conditions previously shown by mass spectrometry to reduce the HA binding groove disulfide bond in CD44 on the surface of cells (Table 1) and in CD44-Fc chimeras which subsequently inhibits HA binding. Cysteines on the surface of CHO-CD44 cells resulting from disulfide bond reduction were alkylated with Dylight-800™ conjugated maleimide allowing reduced proteins to be visualised on a LICOR™ imaging system at 800 nm where proteins containing reduced cysteines show up as green bands (Fig 5C). Control cells were alkylated without prior reduction. The overall level of reduced cysteines is increased after reduction with both TCEP-HCl and Trx1 (Trx1>TCEP-HCl>>control). After scanning, the gel was transferred to nitrocellulose membrane and western blotted for CD44. Upon overlaying the western blot with the gel the red CD44 band overlays with the green bands at around 85kDa, which is the expected mass of the CD44 construct. Other bands containing reduced protein are seen in this assay and increase in intensity upon reduction relative to the non-reduced control and represent other membrane proteins with labile disulfide bonds. To determine if other proteins of similar weight are also being reduced and contributing to the signal in the CD44 region, the lysate was immunoprecipitated with anti-CD44 coated magnetic beads and visualised in the LICOR™ scanner (Fig 5D). Intense green bands at around 85kDa can be seen in both TCEP-HCl and Trx1 reduced CHO-CD44 cells but not in the control cells confirming CD44 is reduced on the surface of CHO-CD44 cells after treatment with both TCEP-HCl and Trx1.

### Reduction of CD44 transfected CHO cells inhibits HA binding

**Fig 6:**
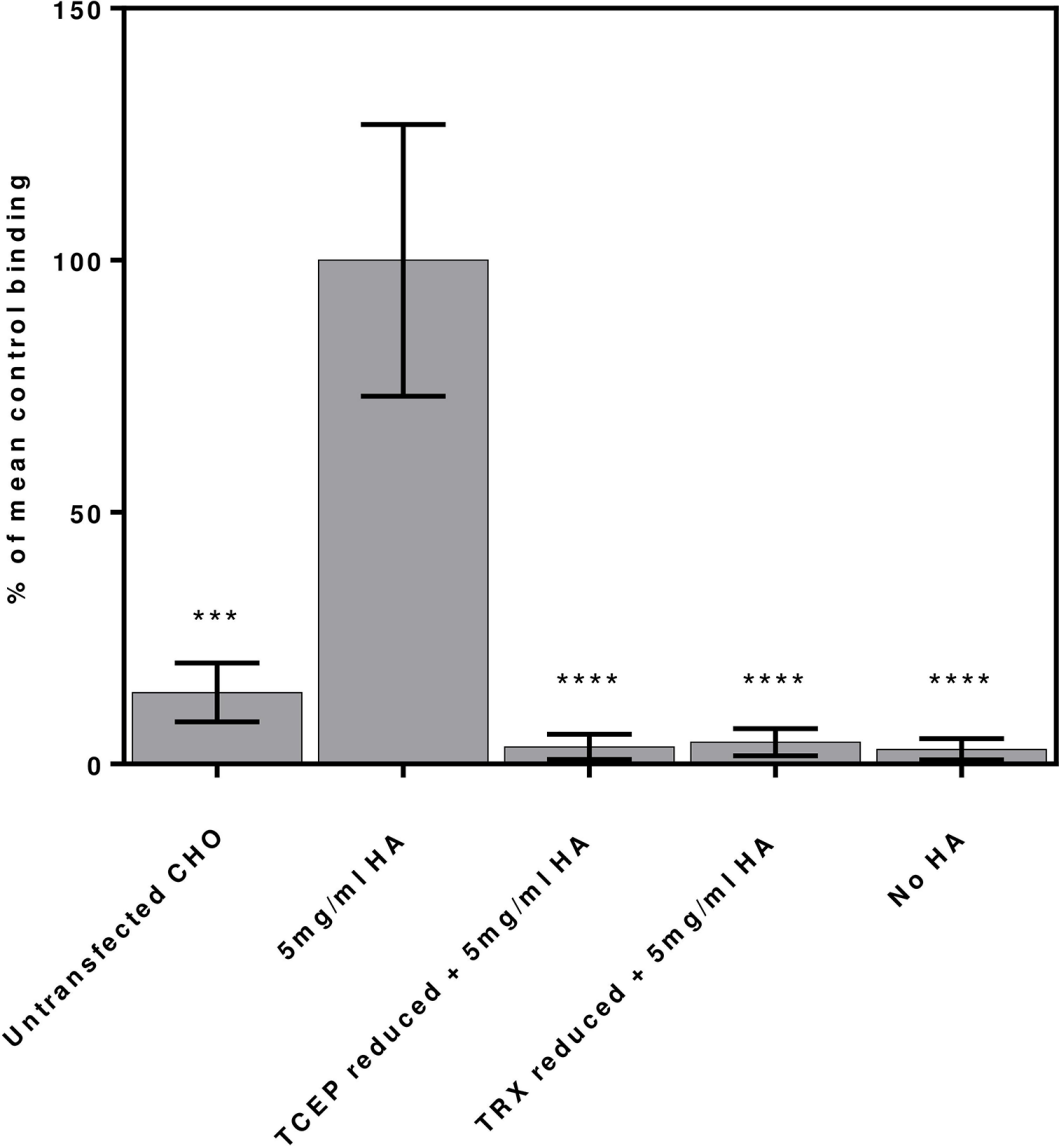
Cell adhesion assay showing the effect of TCEP-HCl and Trx1 reduction of untransfected and hCD44 transfected CHO cells binding to HA coated plates. Binding data are compositions of three independent experiments all normalised assuming non reduced CHO-hCD44 binding to HA coated wells as 100% response. Mean and SEM of total fluorescence are shown. The differences from control: *, P < 0.05; **, P < 0.01; ***, P < 0.001; ***, P < 0.0001

The effect of reducing CD44 on the surface of transfected CHO cells was investigated using a CD44 dependent cell adhesion assay to plate bound HA and is shown in Fig 6. CHO-K1 or CHO-hCD44 cells were stained with calcein and subjected to reduction with either TCEP-HCl or Trx1 and alkylation with NEM. Control CHO-hCD44 cells were alkylated with NEM without prior reduction. hCD44 transfected CHO-K1 cells show a significant increase in binding to HA relative to untransfected CHO-K1 cells. Similarly CHO-hCD44 cells do not bind to wells in the absence of HA, verifying that the binding of CHO-hCD44 cells to HA is dependent upon CD44 cell surface expression. Reduction of CD44 on CHO-hCD44 as described previously inhibits the CD44-HA binding and abolishes adhesion of CHO-hCD44 cells to HA coated plates providing further evidence that reduction can lead to loss of HA binding by CD44 with effects on cell adhesion.

## Discussion

CD44 belongs to a family of HA binding proteins called hyaladherins that contain the link module protein fold. Two disulfide bonds are conserved across the link domain superfamily, one at the core of the link module which stabilises the fold and one which resides in the shallow groove which forms the binding site for HA. Type B link module proteins such as CD44 have N and C terminal extensions to the link module which are stabilised via a third disulfide bond. We have shown that the disulfide bond in the HA binding groove of CD44 (Cys77-Cys97) and not the disulfide bond in the core of the link module (Cys53-Cys108) can be reduced under redox conditions that mimic those found in inflammation.

Mass spectrometry screens identify reduced Cys77-Cys97 on a variety of human and mouse cell lines which had been subjected to reduction by the chemical reductant TCEP, and a spectrum of thiol reductase enzymes such as Trx1. Cys77-Cys97 was also reduced in CD44 purified from human PBMC’s that had been activated by superantigen. Previously, CD44 purified from splenocytes of mice subjected to LPS induced endotoxaemia was deemed to contain a reduced disulfide bond but the identity of the bond was never determined [9]. Detailed kinetic analysis of the rate of reactions of the link domain disulfide bonds in recombinant soluble CD44 confirm that the Cys77-Cys97 disulfide bond is more reactive than Cys53-Cys108 due to its exposed orientation on the surface of CD44 and its reactive–LH hook configuration. The redox state of the third disulfide bond in the link module extension (Cys28-Cys129) could not be determined due to incompatibility of the tryptic peptides which contain the cysteines with the mass spectrometry experiments.

Reduction of the Cys77-Cys97 disulfide bond negatively regulates binding function. Reduced soluble CD44 can no longer bind HA and cells expressing CD44 no longer adhere to HA coated surfaces after treatment with TCEP and Trx1. As the Cys77-Cys97 disulfide bond not only stabilises the HA binding groove but is also in direct contact with bound HA, so reduction of this disulfide bond appears to destabilise both the structure of binding groove and the interaction with HA and thus acts as a redox switch.

There is precedence for such regulation *in vivo*. CD44 is upregulated on the surface of T cells after activation [25], however HA binding function needs to be ‘switched on’. Receptor clustering [26] and relieving steric inhibition of the HA binding site by enzymatic removal of sialic acid moieties capping N-linked glycosylation sites [27,28] have been suggested as mechanisms for induction of HA binding. Once initiated HA binding activity is transient only lasting a few days after activation [29] allowing T cells migrate through the blood circulatory system to sites of inflammation [30]. The loss of HA binding is not due to CD44 down regulation as levels remain high long after the immune challenge has been cleared [31] suggesting that the protein structure and ligand binding is modulated in some way independent upon CD44 expression levels at the cell surface. Inhibition of HA binding by enzymatic reduction of the labile disulfide bond described here by reductases secreted at sites of inflammation could account for the observations.

Redox regulation of proteins through the enzymatic reduction of labile allosteric disulfide bonds is a widespread phenomenon. About 30 proteins have been shown to modulate their function through allosteric switching (reviewed in [12,32]) with many more predicted to contain allosteric disulfide bonds through data mining [23] molecular modelling [33] and experimental screens [9]. A variety of different functions have been shown to be affected by allosteric disulfide bonds in membrane or extracellular proteins. These include integrins where the interaction of platelet integrin αIIbβ3 with fibrinogen is regulated by PDI [34]. Cytokine receptors where CD132, the common gamma chain of the IL-2 family of cytokine receptors contains an allosteric disulfide bond at the interface with bound IL-2 and its redox state controls IL-2 binding and signalling [19]. The T cell surface antigen CD4 has been shown to homo dimerise through an allosteric disulfide bond in domain 2, which alters its function from a co stimulatory molecule [35] to a receptor involved in HIV viral fusion and infection [36]. These studies extend the range of different functions to include an important adhesion protein CD44.

Redox modulation of HA binding in the hyaladherins may be important in regulation of the movement of leukocytes through the lymphatic system during immune responses and inflammation. Conversely as CD44 is a marker for chronic autoimmune disease [37] and plays an important role in cancer metastasis [3] inhibiting the CD44/HA interaction though redox changes offers a mechanism of regulating such processes. Targeting allosteric disulfide bonds has recently been highlighted as a strategy of controlling protein function in disease [38] either by targeting the enzymes involved in the process or the modified protein. CD44 is a particularly attractive target for this approach given its involvement in inflammation and cancer. The ability to regulate the CD44/HA interaction through therapeutic manipulation of the redox state of the Cys77-Cys97 disulfide bond could provide a handle towards limiting leukocyte migration to sites of inflammation in autoimmune disease and the migration of metastatic cancer cells thought the lymphatic system.

## Supporting Information

S1 Table Extracted ion chromatogram peak areas for all of the peptides used in the kinetic trapping experiment. (PDF)
