# Supplementary material for: CD44 Binding to Hyaluronic Acid Is Redox Regulated by a Labile Disulfide Bond in the Hyaluronic Acid Binding Site
Source: PLoS One. 2015 Sep 17;10(9):e0138137. doi: 10.1371/journal.pone.0138137 (PMC4574955; doi:10.1371/journal.pone.0138137)
Supplement: S1 Table — (PDF) [file pone.0138137.s001.pdf]

Experiment	Precursor M/z	Peptide Sequence	Total Extracted Area	Isotope Dot Product	Average Mass Error (PPM)	Label	Normal+hydrolysed areas	NEM:D5-NEM
Control 1	631.29651	ALSIGFE[+22]TC[+143.1]R	703969728	0.9521	-0.1	NEM	76194880	0.05
	624.80692	ALSIGFE[+22]TC[+130.1]R	76194880	0.98	0.1	d5 NEM	1621858944	
	622.29123	ALSIGFE[+22]TC[+125]R	917889216	0.9417	-0.7			
	633.81220	ALSIGFE[+22]TC[+148.09]R	#N/A	0	#N/A			
	620.30554	ALSIGFETC[+143.1]R	19773421568	0.9938	0	NEM	4117216960	0.06
	613.81595	ALSIGFETC[+130.1]R	3381133312	0.992	-0.1	d5 NEM	68952655872	
	611.30026	ALSIGFETC[+125]R	49179234304	0.9936	0.4			
	622.82123	ALSIGFETC[+148.09]R	736083648	0.9955	0.5			
	497.23151	TEAADLC[+143.1]K	8649627648	0.9934	0.3	NEM	4565847680	0.12
	490.74192	TEAADLC[+130.1]K	2812966400	0.9874	0.1	d5 NEM	37936545792	
	488.22623	TEAADLC[+125]K	29286918144	0.9879	0.9			
	499.74720	TEAADLC[+148.09]K	1752881280	0.9425	-0.2			
	626.85253	NQVSLTC[+148.09]LVK	16681157	0.8617	-0.6	NEM	520898181	0.08
	624.33684	NQVSLTC[+143.1]LVK	487053184	0.9768	-0.2	d5 NEM	6287540096	
	617.84725	NQVSLTC[+130.1]LVK	504217024	0.9792	-0.1			
	615.33156	NQVSLTC[+125]LVK	5800486912	0.9913	0.2			
	1115.55143	TPEVTC[+148.09]VVVDVSHEDPEVK	865930560	0.8781	-8.9	NEM	7243879744	0.15
	1113.03574	TPEVTC[+143.1]VVVDVSHEDPEVK	7368753664	0.9971	0	d5 NEM	46881686016	
	1106.54615	TPEVTC[+130.1]VVVDVSHEDPEVK	6377949184	0.9166	2.9			
	1104.03046	TPEVTC[+125]VVVDVSHEDPEVK	39512932352	0.9989	0			
Control 2	631.29651	ALSIGFE[+22]TC[+143.1]R	5162684	0.4835	-3.8	NEM	78364452	0.11
	624.80692	ALSIGFE[+22]TC[+130.1]R	352277664	0.9381	-1.7	d5 NEM	729560672	
	622.29123	ALSIGFE[+22]TC[+125]R	73201768	0.944	-1.8			
	633.81220	ALSIGFE[+22]TC[+148.09]R	377283008	0.9689	-1.1			
	620.30554	ALSIGFETC[+143.1]R	4694376448	0.99	0.7	NEM	10556695552	0.20
	613.81595	ALSIGFETC[+130.1]R	29085110272	0.9972	0.3	d5 NEM	53408198656	
	611.30026	ALSIGFETC[+125]R	5862319104	0.9922	0.8			
	622.82123	ALSIGFETC[+148.09]R	24323088384	0.9935	0.3			

	497.23151	TEAADLC[+143.1]K	3392632832	0.9899	0.5	NEM	14535096320	0.24
	490.74192	TEAADLC[+130.1]K	46457577472	0.9917	1.4	d5 NEM	59946619904	
	488.22623	TEAADLC[+125]K	11142463488	0.986	1.1			
	499.74720	TEAADLC[+148.09]K	13489042432	0.9896	0.7			
	626.85253	NQVSLTC[+148.09]LVK	5769961472	0.9934	0.1	NEM	4633893120	0.44
	624.33684	NQVSLTC[+143.1]LVK	2661230336	0.9581	-2.2	d5 NEM	10534407680	
	617.84725	NQVSLTC[+130.1]LVK	4764446208	0.9938	0.5			
	615.33156	NQVSLTC[+125]LVK	1972662784	0.9499	-3.1			
	1113.03574	TPEVTC[+143.1]VVVDVSHEDPEVK	722293376	0.9974	6.6	NEM	1968385408	0.19
	1115.55143	TPEVTC[+148.09]VVVDVSHEDPEVK	30690064	0.9405	-4.2	d5 NEM	10418448144	
	1106.54615	TPEVTC[+130.1]VVVDVSHEDPEVK	10387758080	0.9773	1.7			
	1104.03046	TPEVTC[+125]VVVDVSHEDPEVK	1246092032	0.9916	0			
TCEP 1	631.29651	ALSIGFE[+22]TC[+143.1]R	10180183	0.9441	-0.4	NEM	220150600	1.31
	624.80692	ALSIGFE[+22]TC[+130.1]R	130853704	0.9158	-1.1	d5 NEM	167969079	
	622.29123	ALSIGFE[+22]TC[+125]R	157788896	0.9416	-0.6			
	633.81220	ALSIGFE[+22]TC[+148.09]R	89296896	0.9496	-0.6			
	620.30554	ALSIGFETC[+143.1]R	1470745984	0.989	0	NEM	7369489792	1.12
	613.81595	ALSIGFETC[+130.1]R	5521117696	0.9932	-0.4	d5 NEM	6584957312	
	611.30026	ALSIGFETC[+125]R	5114211328	0.9921	0.4			
	622.82123	ALSIGFETC[+148.09]R	1848372096	0.9946	0.2			
	497.23151	TEAADLC[+143.1]K	8723124	0.968	0.2	NEM	35256217	0.47
	490.74192	TEAADLC[+130.1]K	28111372	0.9884	-0.2	d5 NEM	75715272	
	488.22623	TEAADLC[+125]K	66992148	0.9837	0.3			
	499.74720	TEAADLC[+148.09]K	7144845	0.8795	0.2			
	624.33684	NQVSLTC[+143.1]LVK	117464088	0.9095	-4.3	NEM	272564604	0.19
	626.85253	NQVSLTC[+143.1]LVK	44543708	0.6276	2	d5 NEM	1403926936	
	617.84725	NQVSLTC[+130.1]LVK	228020896	0.9937	0.2			
	615.33156	NQVSLTC[+125]LVK	1286462848	0.9583	-1.8			
	1113.03574	TPEVTC[+143.1]VVVDVSHEDPEVK	8531346432	0.9973	0.3	NEM	4958519808	0.13

	1115.55143	TPEVTC[+143.1]VVVDVSHEDPEVK	965277952	0.9101	-7.3	d5 NEM	37630845952	
	1106.54615	TPEVTC[+130.1]VVVDVSHEDPEVK	3993241856	0.9442	0.2			
	1104.03046	TPEVTC[+125]VVVDVSHEDPEVK	29099499520	0.9986	0			
TCEP 2	631.29651	ALSIGFE[+22]TC[+143.1]R	741649280	0.9699	-0.5	NEM	1645173888	3.17
	624.80692	ALSIGFE[+22]TC[+130.1]R	265132736	0.9489	-1	d5NEM	518519760	
	622.29123	ALSIGFE[+22]TC[+125]R	903524608	0.9778	-8.7			
	633.81220	ALSIGFE[+22]TC[+148.09]R	253387024	0.9838	-4.3			
	620.30554	ALSIGFETC[+143.1]R	53252493312	0.9926	1	NEM	1.19052E+11	2.44
	613.81595	ALSIGFETC[+130.1]R	33908185088	0.9948	0.8	d5NEM	48872246272	
	611.30026	ALSIGFETC[+125]R	65799614464	0.9925	1			
	622.82123	ALSIGFETC[+148.09]R	14964061184	0.9948	0.2			
	497.23151	TEAADLC[+143.1]K	14068485120	0.9873	0.7	NEM	53395418112	0.99
	490.74192	TEAADLC[+130.1]K	43152801792	0.9891	0.9	d5NEM	54176752640	
	488.22623	TEAADLC[+125]K	39326932992	0.9848	1.4			
	499.74720	TEAADLC[+148.09]K	11023950848	0.9916	0.6			
	624.33684	NQVSLTC[+143.1]LVK	8266940416	0.9906	0	NEM	14739148288	0.53
	626.85253	NQVSLTC[+148.09]LVK	13174813696	0.9944	0.2	d5 NEM	27817507840	
	617.84725	NQVSLTC[+130.1]LVK	14642694144	0.9953	0.5			
	615.33156	NQVSLTC[+125]LVK	6472207872	0.9778	-0.7			
	1113.03574	TPEVTC[+143.1]VVVDVSHEDPEVK	1490005248	0.9908	8.9	NEM	3822074112	0.28
	1115.55143	TPEVTC[+148.09]VVVDVSHEDPEVK	1580609280	0.9912	0	d5 NEM	13672255232	
	1106.54615	TPEVTC[+130.1]VVVDVSHEDPEVK	12091645952	0.9609	2.3			
	1104.03046	TPEVTC[+125]VVVDVSHEDPEVK	2332068864	0.9943	0.6			
Trx1 1	631.29651	ALSIGFE[+22]TC[+143.1]R	58135508	0.9851	-2.3	NEM	199239252	2.07
	624.80692	ALSIGFE[+22]TC[+130.1]R	66937144	0.914	-0.7	d5 NEM	96313804	
	622.29123	ALSIGFE[+22]TC[+125]R	141103744	0.966	-2			
	633.81220	ALSIGFE[+22]TC[+148.09]R	29376660	0.9591	-2.3			
	620.30554	ALSIGFETC[+143.1]R	10507568128	0.9903	0.6	NEM	35237508096	1.73
	613.81595	ALSIGFETC[+130.1]R	14059240448	0.9946	1.1	d5 NEM	20357311488	

	611.30026	ALSIGFETC[+125]R	24729939968	0.9927	0.6			
	622.82123	ALSIGFETC[+148.09]R	6298071040	0.9967	-0.3			
	497.23151	TEAADLC[+143.1]K	7664519	0.96	0.2	NEM	47955575	1.31
	490.74192	TEAADLC[+130.1]K	29647598	0.9851	0.3	d5 NEM	36611061	
	488.22623	TEAADLC[+125]K	40291056	0.983	0.4			
	499.74720	TEAADLC[+148.09]K	6963463	0.9639	-1			
	624.33684	NQVSLTC[+143.1]LVK	268244288	0.9501	-3.7	NEM	5371636032	0.48
	626.85253	NQVSLTC[+148.09]LVK	175721840	0.9604	0	d5 NEM	11239834992	
	617.84725	NQVSLTC[+125]LVK	5103391744	0.9948	-0.6			
	615.33156	NQVSLTC[+130.1]LVK	11064113152	0.9943	-0.2			
	1113.03574	TPEVTC[+143.1]VVVDVSHEDPEVK	1321026048	0.9959	1.3	NEM	4415888896	1.90
	1115.55143	TPEVTC[+148.09]VVVDVSHEDPEVK	679158400	0.979	-0.1	d5 NEM	2329018752	
	1106.54615	TPEVTC[+130.1]VVVDVSHEDPEVK	1649860352	0.992	-0.4			
	1104.03046	TPEVTC[+125]VVVDVSHEDPEVK	3094862848	0.993	-0.3			
Trx1 2	620.30554	ALSIGFETC[+143.1]R	8183165440	0.9813	-0.5	NEM	19804349952	1.88
	613.81595	ALSIGFETC[+130.1]R	6436690944	0.9922	-0.2	d5NEM	10523235584	
	611.30026	ALSIGFETC[+125]R	11621184512	0.9879	-0.3			
	622.82123	ALSIGFETC[+148.09]R	4086544640	0.9927	0.7			
	497.23151	TEAADLC[+143.1]K	1029117440	0.9802	0.4	NEM	5294561280	0.98
	490.74192	TEAADLC[+130.1]K	4384987648	0.9892	0.3	d5NEM	5383402176	
	488.22623	TEAADLC[+125]K	4265443840	0.9874	0.8			
	499.74720	TEAADLC[+148.09]K	998414528	0.9829	0.6			
	624.33684	NQVSLTC[+143.1]LVK	1614599424	0.9157	0.2	NEM	2229929600	0.41
	626.85253	NQVSLTC[+148.09]LVK	4388235776	0.886	0.1	d5 NEM	5396191104	
	617.84725	NQVSLTC[+130.1]LVK	1007955328	0.9714	-1.7			
	615.33156	NQVSLTC[+125]LVK	615330176	0.9541	-1.7			
	1113.03574	TPEVTC[+143.1]VVVDVSHEDPEVK	5194569728	0.9766	13.8	NEM	5994161920	0.12
	1115.55143	TPEVTC[+148.09]VVVDVSHEDPEVK	182898640	0.4028	1.9	d5 NEM	48397877200	
	1106.54615	TPEVTC[+130.1]VVVDVSHEDPEVK	48214978560	0.8854	5			

	1104.03046	TPEVTC[+125]VVVDVSHEDPEVK	799592192	0.9028	0.9			
